# Diffuse myocardial fibrosis in children after heart transplantation

**DOI:** 10.1186/1532-429X-16-S1-P135

**Published:** 2014-01-16

**Authors:** Eugenie Riesenkampff, Steven C Greenway, Paul Kantor, Helen Holtby, Rajiv Chaturvedi, Shi-Joon Yoo, Mike Seed, Andreas Greiser, Lars Grosse-Wortmann

**Affiliations:** 1 Labatt Family Heart Centre in the Department of Paediatrics, Hospital for Sick Children, Toronto, Ontario, Canada; 2 Healthcare Sector, Siemens AG, Erlangen, Germany

## Background

It is unclear if children after heart transplantation (HTX) are at risk for developing increased myocardial fibrosis. Diffuse myocardial fibrosis can be estimated by myocardial longitudinal relaxation (T1) times.

## Methods

Twenty cardiovascular magnetic resonance (CMR) studies in 17 patients after HTX (mean age 13.2 years, range 1.2 - 17.4 years, 9 female) were analysed retrospectively and compared to CMR studies in nine healthy controls (mean age 12.1 years, range 9.2 - 16.7, 4 female). Patients with clinically significant rejection were excluded. T1 measurements were performed at a single mid-ventricular short axis slice orientation before and > 10 minutes after the application of 0.2 mmol/kg gadopentetate dimeglumine (Gd) in the interventricular septum (IVS), LV lateral wall and the complete LV myocardium (Image). The tissue-blood partition coefficient was calculated as a function of the ratio of T1 change of myocardium as compared to blood.

## Results

Pre-contrast T1 times before the application of Gd were significantly higher in HTX patients compared to controls (LV lateral wall 977 ± 40 msec. versus 923 ± 12 msec., p < 0.001; IVS 1008 ± 32 msec. versus 974 ± 21 msec., p < 0.005; complete LV myocardium 992 ± 34 msec. versus 951 ± 16 msec., p < 0.005), whereas the reduced post-contrast T1 times in the HTX patients showed a trend towards being shorter than in controls but failed to reach statistical significance. Tissue-blood partition coefficients were elevated in patients after HTX in the LV lateral wall (0.45 ± 0.06 versus 0.40 ± 0.03, p < 0.01) and the complete LV myocardium (0.47 ± 0.06 versus 0.43 ± 0.03, p < 0.05). The difference in the IVS failed to reach statistical significance (0.48 ± 0.06 versus 0.45 ± 0.03, p = 0.122).

## Conclusions

Diffuse fibrosis is present in children after HTX as evidenced by pre- and post-contrast myocardial T1 mapping. The technique may be suitable for the detection of early signs of adverse remodeling after HTX.

## Funding

This study was partly funded by the Labatt Family Heart Centre, Witchell Fellowship, and by Siemens.

**Figure 1:**
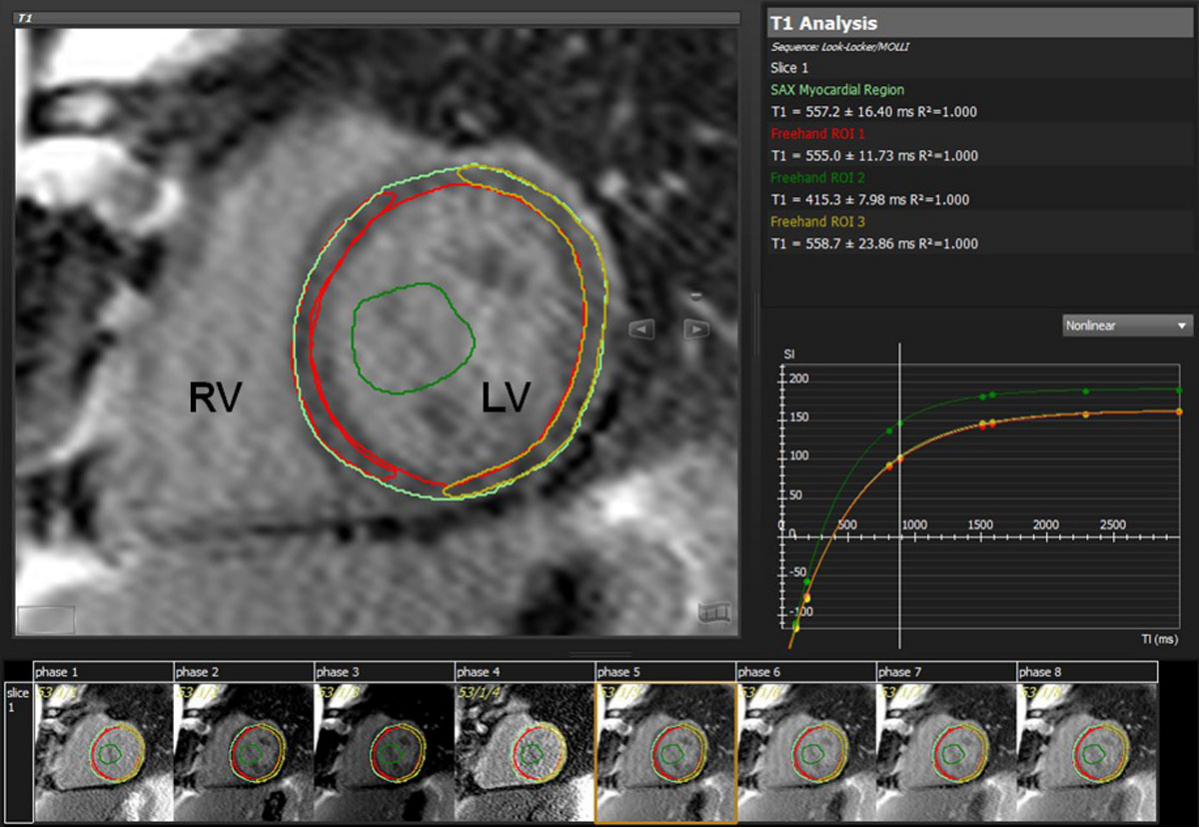
**Tissue longitudinal relaxation (T1) kinetics, derived from a short axis image in a patient after heart transplantation**. LV = left ventricle, RV = right ventricle. Light green = complete LV myocardium, red = septal myocardium, yellow = free wall LV myocardium, dark green = blood pool.

